# A Transposable Element Insertion Confers Xenobiotic Resistance in Drosophila

**DOI:** 10.1371/journal.pgen.1004560

**Published:** 2014-08-14

**Authors:** Lidia Mateo, Anna Ullastres, Josefa González

**Affiliations:** Institute of Evolutionary Biology (CSIC- Universitat Pompeu Fabra), Barcelona, Spain; University of Utah School of Medicine, United States of America

## Abstract

The increase in availability of whole genome sequences makes it possible to search for evidence of adaptation at an unprecedented scale. Despite recent progress, our understanding of the adaptive process is still very limited due to the difficulties in linking adaptive mutations to their phenotypic effects. In this study, we integrated different levels of biological information to pinpoint the ecologically relevant fitness effects and the underlying molecular and biochemical mechanisms of a putatively adaptive TE insertion in *Drosophila melanogaster*: the *pogo* transposon *FBti0019627*. We showed that other than being incorporated into *Kmn1* transcript, *FBti0019627* insertion also affects the polyadenylation signal choice of *CG11699* gene. Consequently, only the short 3′UTR transcript of *CG11699* gene is produced and the expression level of this gene is higher in flies with the insertion. Our results indicated that increased *CG11699* expression leads to xenobiotic stress resistance through increased *ALDH-III* activity: flies with *FBti0019627* insertion showed increased survival rate in response to benzaldehyde, a natural xenobiotic, and to carbofuran, a synthetic insecticide. Although differences in survival rate between flies with and without the insertion were not always significant, when they were, they were consistent with *FBti0019627* mediating resistance to xenobiotics. Taken together, our results provide a plausible explanation for the increase in frequency of *FBti0019627* in natural populations of *D. melanogaster* and add to the limited number of examples in which a natural genetic mutation has been linked to its ecologically relevant phenotype. Furthermore, the widespread distribution of TEs across the tree of life and conservation of stress response pathways across organisms make our results relevant not only for Drosophila, but for other organisms as well.

## Author Summary

Given the predictions of future environmental fluctuations, it is crucial to understand how organisms adapt to changing environments. The fruit fly *Drosophila melanogaster* is an ideal model organism to study environmental adaptation because of our deep understanding of developmental, physiological, and metabolic networks, as well as the ease of experimental manipulation. In this study, we showed that a previously identified putatively adaptive mutation, the insertion of the transposable element *FBti0019627*, mediates resistance to both natural and synthetic xenobiotics in *Drosophila melanogaster*. By combining experimental and computational approaches, we further elucidated the molecular and the biochemical mechanisms underlying this natural adaptive mutation. Our results should be relevant for other organisms as well since there are many similarities between species in the way cells respond to stress.

## Introduction

Understanding the functional consequences of naturally occurring mutations is one of the key challenges in modern biology. Recent years have seen an explosion in the availability of genomic data that have opened up the possibility of searching for adaptive mutations on an unprecedented scale [1]. Although there are some examples in which adaptive mutations have been connected to their phenotypic effects [2]–[5], our knowledge of the functional consequences of particular genetic variants is still very limited. Mapping genotype to phenotype is a difficult task due to the large number of genes that contribute to some phenotypes, to the pervasiveness of genetic interactions, and to the complex environmental influences on the phenotypic outcome [6], [7].

Current efforts in genotype-phenotype mapping include projects in several model organisms [8]. Among them, *Drosophila melanogaster* is one of the most promising cases due to the high quality gene annotation, deep understanding of developmental, physiological, and metabolic networks, and the availability of genetic resources. Because genes tend to work in evolutionarily conserved pathways, genotype-phenotype insights obtained in *D. melanogaster* provide valuable information that is relevant for other organisms as well [7]. Most ongoing projects in Drosophila focus on mapping SNP variants to a given set of phenotypic traits such as olfactory behavior or stress resistance [9]–[12]. While SNPs certainly contribute to ecologically relevant phenotypes, these efforts ignore other types of mutations, such as those caused by transposable element (TE) insertions.

TEs have the ability to generate mutations of great variety and magnitude, ranging from subtle regulatory mutations to large genomic rearrangements that can have complex phenotypic effects. Additionally, TEs have been shown to be susceptible and responsive to environmental changes; as such, they might have an important role in environmental adaptation [13]–[15]. We have recently used TEs as a tool to identify putatively adaptive mutations to the out-of-Africa environments in *D. melanogaster* on a genome-wide scale [16], [17]. We screened 763 TEs and identified 18 putatively adaptive TEs based on their population dynamics [16], [18]. For a subset of the candidate TEs, we also demonstrated that they show signatures of selective sweeps [16], [19], evidence of population differentiation [17], and two of them, *FBti0019430* and *FBti0018880*, have already been linked to adaptive fitness effects [20]–[22]. Thus, putatively adaptive TEs in this set are good candidates to perform follow-up experiments that should allow us to map genotype to phenotype and to identify the underlying mechanisms of adaptive mutations.

**Figure 1:**
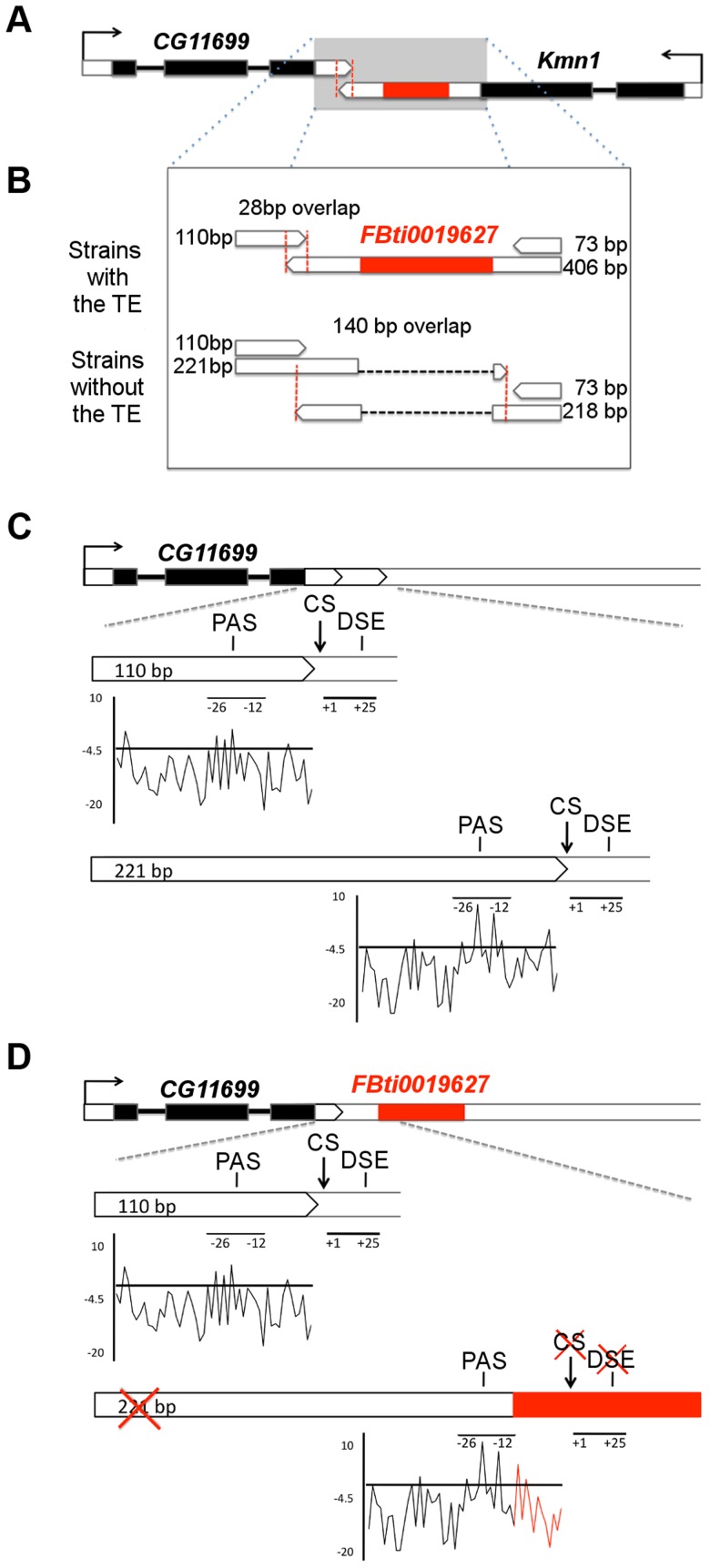
*FBti0019627* is inserted in the 3′ UTR of *Kmn1* and affects the length of *Kmn1* and *CG11699*. (A) Genomic location of *FBti0019627* insertion. Exons are depicted as black boxes, UTRs as white boxes and the TE as a red box. The arrows indicate the direction of gene transcription. Red vertical dashed lines delimit the overlap region between the two genes. (B) 3′ UTRs of *CG11699* and *Kmn1* transcripts in flies with and without *FBti0019627* insertion. Size of the different 3′UTRs and overlap region between the two genes is indicated. (C) Localization of the PolyAdenylation Signal (PAS), cleavage site (CS) and GU rich Downstream Sequence Element (DSE) for the two *CG11699* transcripts in flies without the insertion. A graphical representation of the scores obtained for the PAS are also given. (D) Localization of the PAS, CS and DSE in flies with the insertion and graphical representation of the scores obtained for the PAS. *FBti0019627* is inserted only 7 bp downstream from the distal PAS and 12 bp upstream the distal CS.

In this study, we focused on mapping one of the previously identified putatively adaptive insertions, the 186 bp *POGON1* element *FBti0019627*, to its ecologically relevant phenotype. *FBti0019627* is inserted in the 3′ UTR region of *kinetochore Mis12-Ndc80 network component 1* (*Kmn1*) gene, and it is closely located to *CG11699*, a gene of unknown function (Figure 1A) [23]. *Kmn1* and *CG11699* genes partially overlap and encode cis-natural antisense transcripts [24]. *FBti0019627* has recently increased in frequency in out-of-African populations most likely due to positive selection, as suggested by the signatures of a selective sweep in the flanking regions of this TE, including *CG11699* and *Kmn1* coding sequences [16]. Here, we used an integrative approach, that combines gene structure and gene expression analyses, protein modeling and docking simulations, enzymatic activity and stress resistance assays, to map genotype to phenotype while disentangling the molecular and biochemical mechanisms underlying the adaptive effect of *FBti0019627* insertion. We show that, besides being incorporated into *Kmn1* transcript, *FBti0019627* affects the choice of polyadenylation signal of *CG11699* and as a result, only the short 3′ UTR transcript of this gene is produced. These structural changes are associated with increased *CG11699* expression in flies with the insertion, leading to xenobiotic resistance through increased *ALDH-III* activity. Xenobiotic resistance is an ecologically relevant phenotypic trait that provides a plausible explanation for the recent increase in the frequency of *FBti0019627* insertion due to positive selection [16], [17].

## Results

### *FBti0019627* affects the structure of two nearby genes

To confirm that the TE is inserted in the *Kmn1* transcript (Figure 1A), we performed 3′ RACE experiments in flies from outbred-1 populations with and without *FBti0019627* insertion (see Material and Methods). As expected, we found that the TE is incorporated into the *Kmn1* transcript in flies with the insertion, while flies without the insertion have a 188 bp shorter transcript due to the absence of the TE (Figure 1B). Additionally, we discovered a previously unreported transcript with a much shorter 3′ UTR, only 73 bp long, that is present both in flies with and without the insertion (Figure 1B).

To check whether the TE also affects the structure of *CG11699*, we carried out 3′ RACE experiments. We found that while flies with *FBti0019627* insertion have only one transcript with a 110 bp long 3′ UTR, flies without the insertion have two transcripts that differ in the length of their 3′ UTRs: 110 bp long and 221 bp long (Figure 1B). We further analyzed whether the difference in *CG11699* transcripts present in flies with and without the insertion is due to *FBti0019627* insertion. We identified the cleavage site of each transcript and performed a motif search analysis to identify the polyadenylation signals (PASs) and GU-rich downstream sequence element (DSEs) that are most likely being used to generate the short and the long 3′ UTR transcripts [25]. We found a weak proximal PAS and a strong distal PAS and their corresponding DSEs upstream and downstream respectively of the two cleavage sites (Figure 1C). In flies with the insertion, the TE is inserted between the PAS and the distal cleavage site disrupting the DSE (Figure 1D). In flies with the insertion, the distal cleavage site is not used; as a consequence, only the transcript with the short 3′ UTR is produced.

Because flies with and without the insertion differ in *CG11699* transcript isoforms, the length of the overlapping region between this gene and *Kmn1* is also different: 28 bp in flies with the insertion and 140 bp in flies without the insertion (Figure 1B). Overall, our results indicated that, besides being incorporated into *Kmn1* transcript, *FBti0019627* insertion affects the PAS choice of *CG11699*. As a result, flies with and without this insertion differ in their *CG11699* transcript isoforms and in the length of the overlap between *CG11699* and *Kmn1*. We decided to focus on *CG11699* for further investigation.

### *FBti0019627* affects the relative abundance of transcript isoforms and increases the total level of expression of *CG11699*

To confirm that only the short 3′ UTR transcript is produced in flies with the insertion, and to determine the relative abundance of the short and long 3′ UTR transcripts in flies without the insertion, we performed transcript-specific qRT-PCR in flies from outbred-1 populations (see Material and Methods). We found that in flies with the insertion, the long 3′ UTR transcript is barely detectable. This confirmed that when the TE is present, only the short 3′ UTR transcript is produced (t-test p-value = 0.003 and 0.004 male and female respectively, Figure S1). On the other hand, in flies without the insertion ∼70% of the total *CG11699* expression is due to the longer transcript (t-test p-value = 0.008 and 0.013 male and female respectively, Figure S1). These results are in accordance with the computational prediction of the distal PAS being stronger than the proximal PAS (Figure 1C).

**Figure 2:**
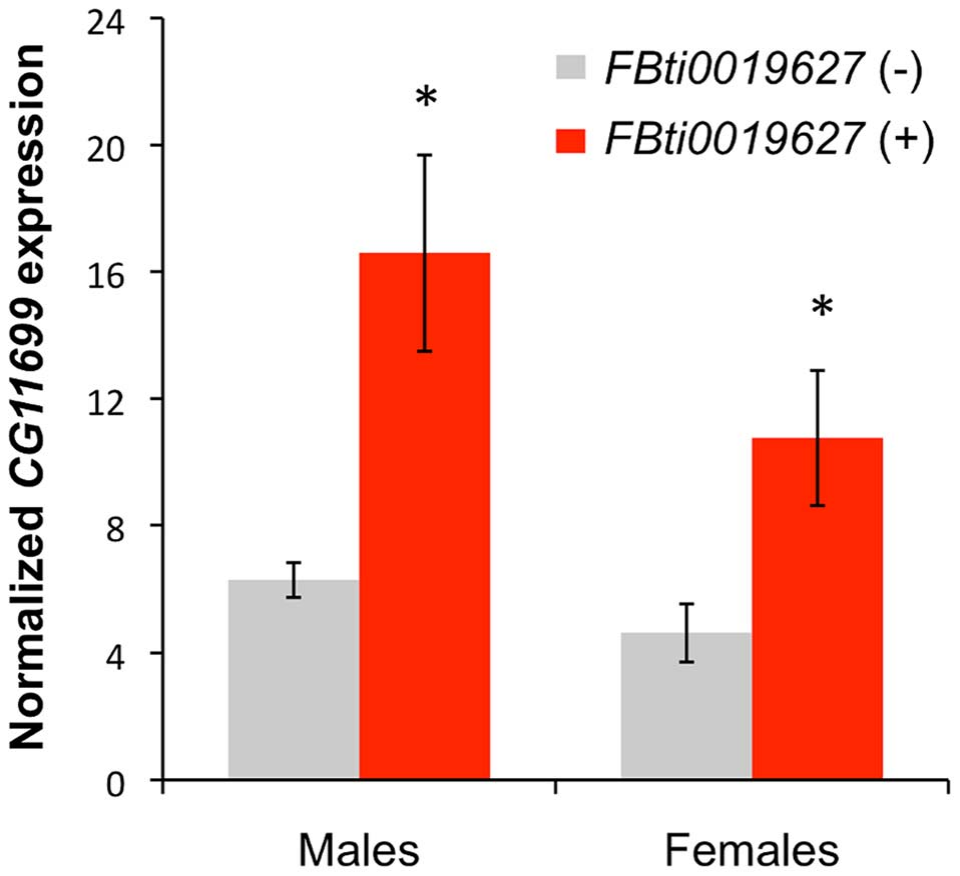
Flies with *FBti0019627* insertion show increased *CG11699* expression. Real-Time PCR quantification of *CG11699* transcript levels in flies without the *FBti0019627* insertion (gray) and with the insertion (red) are shown for males and females. Average copy number of *CG11699* relative to *Act5C* with error bars representing the S.E.M for three biological replicas are given.

Short 3′UTR transcript isoforms usually show increased relative expression levels compared to long 3′UTR transcript isoforms [26], [27]. Thus, we expected that flies with the insertion would have a higher level of expression of *CG11699* compared to flies without the insertion, because 100% of the *CG11699* isoforms are short in flies with the insertion, while only 30% of the isoforms are short in flies without the insertion. Indeed, our results showed that flies with the insertion have an increased level of expression of *CG11699*: ∼2.6 fold (t-test p-value = 0.011) in males and ∼2.3 in females (t-test p-value = 0.029) (Figure 2). Thus, *FBti0019627* insertion affects the relative abundance of the short and long 3′ UTR transcripts and it is also associated with an overall increased expression of *CG11699*.

### *FBti0019627* is associated with increased *ALDH-III* activity

**Figure 3:**
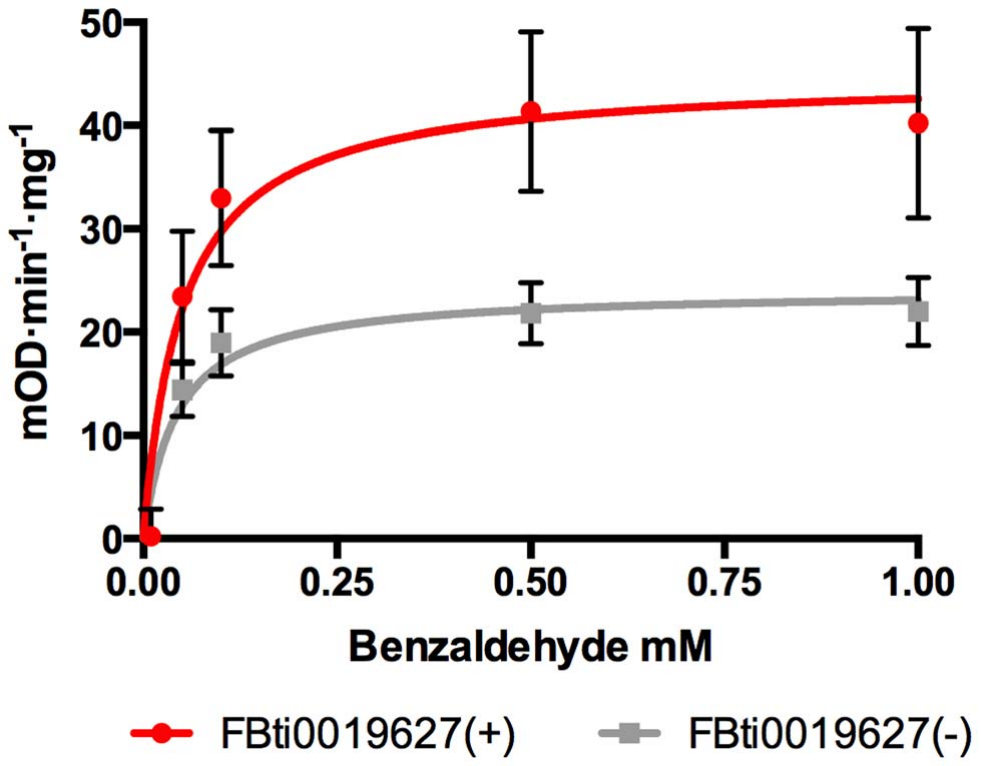
Flies with *FBti0019627* insertion show increased *ALDH-III* activity. Substrate-velocity curves for *ALDH-III* activity in flies with (red line) and without (grey line) *FBti0019627* insertion. Each data point is the average reaction rate of three biological replicates with corresponding standard error bars. Vmax (95% confidence interval) is 44.73 mOD·min^−1^·mg^−1^ (32.15 to 57.32 mOD·min^−1^·mg^−1^) for flies with *FBti0019627* insertion and 24.06 mOD·min^−1^·mg^−1^ (18.65 to 29.48 mOD·min^−1^·mg^−1^) for flies without the insertion.

*CG11699* encodes a transmembrane protein of unknown function that physically interacts with *Aldehyde dehydrogenase III* (*ALDH-III*) [28]. It has been shown that over-expression of *CG11699* increases *ALDH-III* activity in phosphorylated membrane extracts [29]. We hypothesized that flies with *FBti0019627* insertion, which have increased *CG11699* expression, would have increased *ALDH-III* activity in the membrane. To test this hypothesis, we measured *ALDH* activity using different concentrations of benzaldehyde, which is a highly reactive substrate of this enzyme (see Material and Methods; [29], [30]). We compared *ALDH-III* substrate-activity curves in flies with and without the insertion from the outbred-1 populations. In agreement with our expectations, we found that flies with the insertion have significantly higher *ALDH-III* enzymatic activity than flies without the insertion (p-value = 0.0042) (Figure 3).

### *FBti0019627* confers resistance to high doses of benzaldehyde

**Figure 4:**
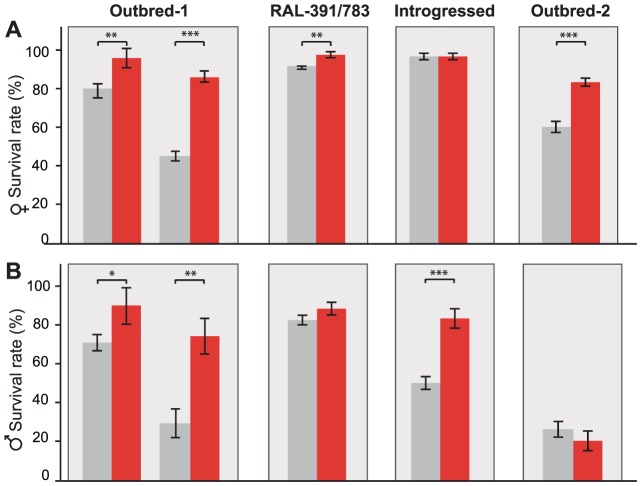
*FBti0019627* is associated with increased resistance to benzaldehyde. Average survival rate of females (A) and males (B) flies from outbred-1 population, DGRP strains (RAL-391 and RAL-783), introgressed strains, and outbred-2 population, after an acute exposure to benzaldehyde. Error bars indicate the S.E.M of the different replicas performed.

Benzaldehyde is highly toxic when present at concentrations that are too high to be rapidly eliminated because it readily form adducts with DNA, RNA, and proteins [31]. Additionally, benzaldehyde generates reactive oxygen species (ROS) that induce lipid peroxidation in the membrane [32]. *ALDH-III* not only metabolizes exogenous aldehydes, such as benzaldehyde, but also plays a protective role against endogenous aldehydes generated as a result of lipid peroxidation [30], [33]. Therefore, flies with *FBti0019627* insertion that show increased *ALDH-III* activity (Figure 3) should be more resistant to high doses of benzaldehyde. To test this hypothesis, we compared the survival rate of outbred-1 populations with and without *FBti0019627* insertion after an acute exposure to benzaldehyde (see Material and Methods). We analyzed 3 replicas of 50 flies each per sex and per strain for unstressed and stressed conditions (1,200 flies total). While there were no differences in survival rate between flies with and without the insertion in unstressed conditions, we found that flies with the insertion showed increased survival rate compared to flies without the insertion when exposed to high concentrations of benzaldehyde (females t-test p-value = 0.0035, odds-ratio (95% confidence intervals) = 3.11 (1.48–6.50), and males t-test p-value = 0.026, odds-ratio = 4.48 (2.24–8.96); Figure 4). We confirmed these results by replicating the experiment using a larger sample size (9 replicas of 50 flies each: 3,600 flies in total) (Figure 4). Again, both male and female flies with the insertion were more resistant to benzaldehyde than flies without the insertion (females t-test p-value≪0.001, odds-ratio = 7.53 (5.42–10.46) and males t-test p-value = 0.0017 odds-ratio = 6.94 (5.17–9.31); Figure 4). These results suggest that *FBti0019627* mediates resistance to benzaldehyde, which is consistent with increased *CG11699* expression (Figure 2) and increased *ALDH-III* activity (Figure 3) observed in flies with this insertion.

To further confirm that resistance to benzaldehyde is due to the insertion and not to any other background mutation present in the outbred-1 populations, we performed the acute exposure to benzaldehyde experiment using flies with three different genetic backgrounds: two DGRP inbred strains, two introgressed strains, and outbred-2 populations (see Material and Methods). We found that females with the insertion showed a significant increase in survival rate compared to females without the insertion in two of the three new backgrounds analyzed: the DGRP strains (Mann-Whitney p-value = 0.001 odds ratio = 8.04 (4.42–14.63)) and the outbred-2 populations (t-test p-value≪0.001 odds ratio = 3.29 (2.14–5.05); Figure 4A). Both introgressed females with and without the TE were highly resistant to benzaldehyde and did not show statistically significant differences (Mann-Whitney, p-value>0.05) (Figure 4A). Similar results were obtained for males; DGRP males with the insertion showed a higher survival rate compared to males without the insertion, although the difference was not statistically significant (t-test, p-value = 0.12). Introgressed males with the insertion were more resistant to benzaldehyde than introgressed males without the insertion (t-test, p-value≪0.001 odds-ratio = 6.94 (3.08–7.06)). Finally, outbred-2 males with and without the insertion were both highly sensitive to benzaldehyde (t-test, p-value>0.05). While the differences in survival rate between flies with and without the insertion were not always significant, when they were, they were consistent with our expectations. These results strongly suggest *FBti0019627* insertion mediates resistance to benzaldehyde and that mutations other than the *FBti0019627* insertion also affect this phenotype.

### *FBti0019627* confers resistance to a carbamate insecticide

Aldehydes are present in decomposing fruits, a common food source for *D. melanogaster* in nature [34]. However, it is not clear whether flies in nature are exposed to such high concentrations of aldehydes as we used in our acute exposure experiments [35]. We searched for other ecologically relevant compounds for *D. melanogaster* natural populations that could also interact with *ALDH-III*.

**Figure 5:**
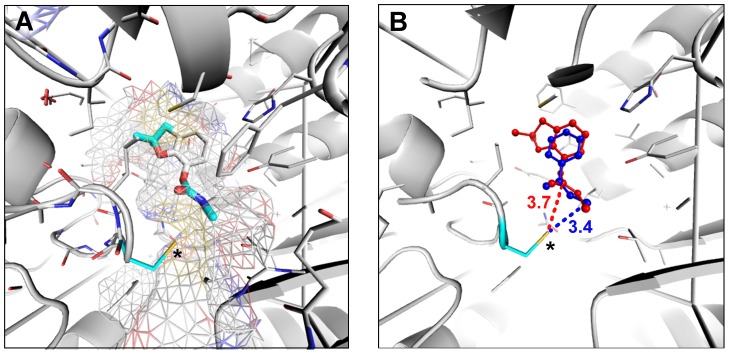
Carbofuran might be an *Aldh-III* inhibitor. (A) The surface of *ALDH-III* catalytic funnel is represented as a mesh with carbon atoms colored in white, nitrogen atoms in blue, oxygen atoms in red, and sulfur atoms in yellow. The docked structure of carbofuran is represented with thicker sticks. The nucleophilic thiol group of the active site cysteine is indicated by an asterisk. (B) The best docked pose of carbofuran and *aldi1* are shown in red and blue, respectively. Note that the aromatic rings and the oxo groups of both compounds are located in the same regions. The distance of the carbamate electrophilic carbon and the distance of the vinyl ketone of *aldi1* to the nucleophilic thiol group of the active site cysteine are given.

Insecticides and herbicides such as carbamates and thiocarbamates are known to inhibit *ALDH2* in humans and rats by covalent modification of the nucleophilic active site residue [36], [37]. *ALDH* enzymes share a wide range of common physiological functions and substrates and are predicted to have very similar catalytic site structures [37], [38]. It is thus possible that carbofuran, a carbamate insecticide, could also react with the active site of *D. melanogaster ALDH-III* inhibiting this enzyme. We built a homology-based model of this protein and we performed preliminary docking studies with *aldi1*, a known *ALDH-III* inhibitor, and with carbofuran. We found that the size and the shape of carbofuran molecule fits in the catalytic funnel of *ALDH-III* (Figure 5a). The aromatic rings and the oxo groups of both compounds are located in the same regions (Figure 5b) and the distance between the electrophilic group of carbofuran and the nucleophilic active site residue of *ALDH-III* is similar to the distance found for the known inhibitor (Figure 5B). Therefore, these preliminary docking results are compatible with carbofuran being a possible *ALDH-III* inhibitor.

**Figure 6:**
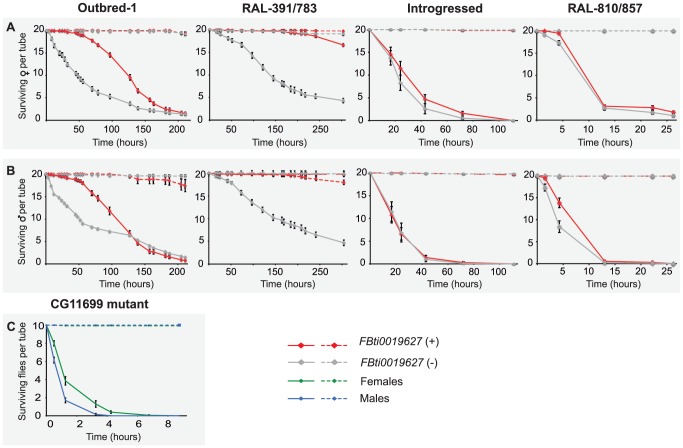
*FBti0019627* is associated with increased resistance to carbofuran. Survival curves of female (A) and male (B) flies from the four different genetic backgrounds analyzed. Solid lines represent survival curves of flies exposed to carbofuran and dashed lines correspond to survival curves of flies in control conditions. Each data point represents the average of surviving flies of 20 replicas of 20 individuals each with error bars indicating the S.E.M. (C) Survival curves for *CG11699* mutant flies exposed to carbofuran (solid lines) and for flies kept in control conditions (dashed lines).

Although we cannot conclude that carbofuran is an *ALDH-III* inhibitor, increased *ALDH-III* activity could also lead to increased carbofuran resistance because carbofuran is an electrophilic molecule that causes lipid peroxidation through the generation of reactive oxygen species (ROS) [39]–[41]. As we have previously mentioned, *ALDH3* is known to efficiently metabolize lipid peroxidation derived aldehydes [30] and could therefore play a protective role against carbofuran toxic effects. We hypothesized that flies with the insertion, which show increased *ALDH-III* activity, could have increased resistance to this carbamate insecticide. In order to evaluate this hypothesis, we compared the survival curves of flies with and without the insertion (3,200 flies in total) that were exposed to concentrations of carbofuran similar to those used in the field (http://www.epa.gov/oppsrrd1/REDs/carbofuran_red.pdf). We used flies with four different genetic backgrounds: outbred-1 populations, DGRP strains and introgressed strains previously used for the benzaldehyde experiments, and two new DGRP strains (see Material and Methods). We found that both males and females flies with the insertion were more resistant to carbofuran than flies without the insertion (Figure 6) (Table 1). Only introgressed males with and without the insertion did not show differences in survival rate (Figure 6B) (Table 1). The magnitude of the effect varied across backgrounds: the effect size was bigger for outbred-1 and RAL-391/783 compared to introgressed and RAL-810/857 (Table 1) strongly suggesting that mutations other than *FBti0019627* influence this phenotype.

**Table 1:** Log-rank test p-value and effect size of the mutation on carbofuran resistance for the four different backgrounds analyzed.

Genetic background	Sex	p-value	odds-ratio (95% confidence interval)
Outbred (1)	female	≪0.001	24.5 (14.3–41.9)
	male	<0.001	12.4 (8.2–18.8)
RAL-391/RAL-783	female	≪0.001	83.1 (33.6–205)
	male	≪0.001	67 (29.2–153.6)
Introgressed	female	≪0.001	1.91 (1.28–2.83)
	male	>0.05	-
RAL-810/RAL-857	female	0.002	1.56 (1.52–2.32)
	male	≪0.001	4.4 (2.8–6.9)

Finally, we also expect that *CG11699* mutant flies, which have been previously shown to be highly sensitive to high doses of benzaldehyde [29], should also be highly sensitive to carbofuran. These mutant flies showed reduced or null *CG11699* expression levels [42], and thus reduced *ALDH-III* activity [29]. As expected, our results showed that all *CG11699* mutant flies died in the first 7 hours of treatment confirming the predicted high sensitivity of these flies to the insecticide (Figure 6).

Our results obtained from four different genetic backgrounds showed that *FBti0019627* insertion mediates resistance to carbofuran insecticide, which is consistent with increased *CG11699* expression (Figure 2) leading to increased *ALDH-III* activity (Figure 3). Similar to the results obtained with benzaldehyde, we also found differences in the magnitude of the effect between backgrounds; this is most likely explained by the contribution of other mutations to this phenotype. Additionally, results obtained with *CG11699* lab mutants further confirmed the association between *CG11699* expression levels, *ALDH-III* activity levels, and xenobiotic resistance.

### *FBti0019627* is not associated with resistance to oxidative stress induced by H_2_O_2_

**Figure 7:**
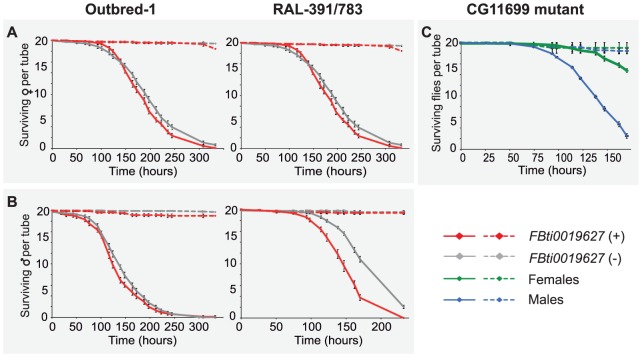
*FBti0019627* is not associated with increased resistance to H_2_O_2_ induced oxidative stress. Survival curves of flies with *FBti0019627* insertion (red) and without the insertion (gray) for female (A) and male (B) flies from outbred-1 population and DGRP strains (RAL-391 and RAL-783). Solid lines represent survival curves of flies exposed to H_2_O_2_ and dashed lines correspond to survival curves of flies in control conditions. Each data point represents the average of surviving flies of 20 replicas of 20 individuals each with error bars indicating the S.E.M. (C) Survival curves for *CG11699* mutant flies exposed to H_2_O_2_ (solid lines) and for flies kept in control conditions (dashed lines).

In Drosophila, there is a common oxidative stress response and a specific oxidative stress response that varies depending on the oxidative stress-inducing agent [43]. Both benzaldehyde and carbofuran are lipophilic electrophiles that induce the generation of reactive oxygen species (ROS) leading to lipid peroxidation [31], [39]–[41]. To test whether *FBti0019627* insertion confers resistance to other oxidative stress-inducing agents with different physicochemical properties than carbofuran and benzaldehyde, we used H_2_O_2_ to induce oxidative stress. While both carbofuran and benzaldehyde are lipophilic compounds, H_2_O_2_ is a small polar molecule that is not expected to directly interact with membranes [44]. We compared the survival curves of outbred-1 populations and DGRP strains with and without the insertion by analyzing 20 replicas of 20 flies each per sex and per strain, for unstressed and stressed conditions (3,200 flies in total). Female outbred-1 flies with the insertion were more sensitive than females without the insertion (log-rank p-value = 0.001, odds-ratio = 1.4 (1–1.8)) while males with the insertion were more resistant (log-rank p-value = 0.019, odds-ratio = 1.5 (1.1–2) (Figure 7). In both cases, the lower confidence interval of the odds-ratio was 1 or close to 1 indicating that these results barely reach statistical significance (see Material and Methods). On the other hand, DGRP strains with the insertion were more sensitive to H_2_O_2_ than strains without the insertion (log-rank p-value≪0.001, both for male and female flies) (Figure 7). However, this result is explained by the presence in RAL-783 of a TE insertion named *Bari-Jheh* that confers resistance to oxidative stress [22]. Flies with *FBti0019627* insertion were equally or more sensitive to H_2_O_2_ compared to flies without the insertion, suggesting that *FBti0019627* does not play a role in resistance to H_2_O_2_ (Figure 7).

If the functional interplay of *CG11699* and *ALDH-III* plays a role in general response to oxidative stress, we would expect *CG11699* mutant flies to be highly sensitive to oxidative stress induced by H_2_O_2_. However, after 138 hours of treatment, 50% of the mutant males and 90% of the mutant females were alive (Figure 7C). These results contrast with the high sensitivity of *CG11699* mutant flies to carbofuran: all flies were dead after only 7 hours of stress exposure (Figure 6C). Taken together, our results indicate that resistance to benzaldehyde and carbofuran in flies with the insertion is due to a specific oxidative stress response induced by lipophilic electrophiles and mediated by *ALDH-III*.

## Discussion

### *FBti0019627* insertion mediates resistance to xenobiotics

In this study, we showed that *FBti0019627* insertion mediates resistance to xenobiotics by increasing *CG11699* expression leading to increased *ALDH-III* activity (Figure 2 and Figure 3). Flies with *FBti0019627* insertion show increased survival in response to benzaldehyde (Figure 4) and to carbofuran (Figure 6) compared to flies without the insertion. Benzaldehyde is an aromatic aldehyde found in fruits in decomposition, and carbofuran is a carbamate insecticide that has been widely used in nature [41]. Thus, both fatty and aromatic aldehydes and carbamate insecticides found in *D. melanogaster* habitats are likely agents of selection driving the previously reported increase in *FBti0019627* frequencies [16], [17]. Note that other *ALDH-III* substrates present in natural *D. melanogaster* habitats could also be acting as agents of selection of this mutation.

We confirmed that xenobiotic resistance is due to *FBti0019627* insertion and not to any other background mutation by performing experiments using flies with five different genetic backgrounds: two pairs of outbred populations, two pairs of DGRP inbred strains, and one pair of introgressed strains. Although outbred populations, inbred strains, and introgressed strains differ in their patterns of linkage disequilibrium, in the composition and site frequency distribution of alleles, and in the presence/absence of heterozygous individuals, we consistently observed that flies with the insertion showed increased resistance to xenobiotics compared to flies without the insertion (Figure 4 and Figure 6). Differences in survival rate between flies with and without the insertion were not always significant. However, when they were, they were consistent with our expectations, suggesting that *FBti0019627* mediates resistance to xenobiotics. The lack of consistent patterns among backgrounds when a different selective agent was used, *i.e.* oxidative stress induced by H_2_O_2_, further reinforces the role of *FBti0019627* in xenobiotic resistance. Effect size of the mutation also varied across backgrounds indicating that genes other than the one affected by the TE insertion are also contributing to the xenobiotic resistance phenotype. These results contrast with previous findings in which the putatively causative mutations of several quantitative traits could not be replicated between strains [12]. While epistatic interactions do not appear to dominate the effect of *FBti0019627*, they probably play an important role.

Although there are a few examples of TE insertions mediating insecticide resistance in Drosophila [20], [22], [45]–[48], previous evidence linking TEs and resistance to natural xenobiotics was only indirect, *i.e.* based on the observation that TEs are enriched within or close to resistance genes [49], [50]. Therefore, our results provide the first experimental evidence for a role of TEs in both natural and synthetic xenobiotic resistance in eukaryotes.

Given the widespread distribution of TEs across the tree of life, the conservation of stress response pathways across organisms, and the ubiquitous presence of natural and/or synthetic xenobiotics in the environment, it is likely that TEs are involved in resistance to xenobiotic stress in organisms other than *D. melanogaster*.

### Changes in alternative polyadenylation of *CG11699* underlie the adaptive effect of *FBti0019627* insertion

Our results indicate that the insertion of *FBti0019627* interferes with the choice of *CG11699* polyadenylation signal (PAS). As a result, in flies with the insertion, only the short 3′ UTR transcript is produced. As expected, the change in the length of the 3′ UTR leads to increased *CG11699* expression levels. Shorter 3′ UTRs isoforms are less likely to possess microRNA binding site and/or other regulatory sequences such as AU-rich elements; consequently, they produce higher levels of transcripts and of protein [26], [27], [51].

Alternative polyadenylation, which leads to transcripts with 3′ UTRs of different lengths, is emerging as a major player in controlling gene regulation [27]. Deciphering the mechanisms behind the choice of alternative polyadenylation sites is considered to be one of the most interesting questions that remains to be answered. Our results provide evidence for TEs playing a role in this selection.

*FBti0019627* insertion also affects the transcript length of *Kmn1*, which could lead to a change in the level of expression of this gene. Further experiments should help elucidate the effect of *FBti0019627* on *Kmn1*, which would provide a more complete picture of the effect of this insertion.

### *Aldh-III* plays a role in xenobiotic resistance in *D. melanogaster*

Besides elucidating the molecular mechanism underlying the adaptive effect of *FBti0019627* insertion, in this analysis we also shed light on its biochemical underpinnings. We showed that increased *CG11699* expression is associated with increased *ALDH-III* activity as was first proposed by Arthaud et al (2011) [29]. Flies with *FBti0019627* insertion are more resistant to benzaldehyde (Figure 4) and carbofuran (Figure 6) but not to H_2_O_2_ (Figure 7) suggesting that resistance to benzaldehyde and carbofuran is due to a specific stress response induced by lipophilic electrophiles and mediated by *ALDH-III*. We also found that a lab mutant strain with null or low levels of *CG11699* expression, which has been previously shown to be sensitive to benzaldehyde [29], is also highly sensitive to carbofuran but not to H_2_O_2_. This result reinforces the functional interplay between *CG11699* expression, *ALDH-III* activity, and xenobiotic resistance. Insecticide resistance is an ongoing challenge for pest management and our results add *ALDH-III* to the list of previously reported enzymes that play a role in this resistance [49].

### TEs as a tool to map genotype to phenotype in *D. melanogaster*

**Figure 8:**
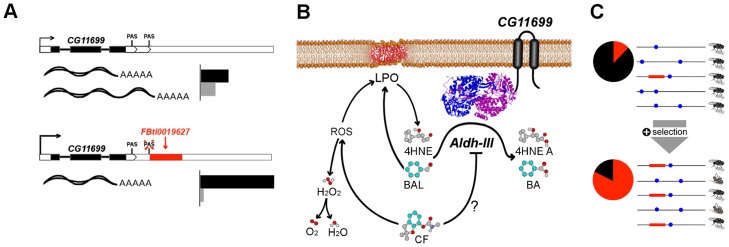
Graphical summary of the results. (A) *FBti0019627* affects *CG11699* PAS choice and, as a consequence, the relative abundance of *CG11699* transcripts changes, and the overall expression of the gene increases (see Figure 1 legend). (B) *CG11699* is a transmembrane protein that physically interacts with *ALDH-III*. Increased *CG11699* expression is associated with increased *ALDH-III* activity that results in resistance to benzaldehyde and to carbofuran. Benzaldehyde (BAL) is oxidized by *ALDH-III* to benzoic acid (BA). Carbofuran (CF) could be inhibiting *ALDH-III*, as suggested by preliminary docking studies, and/or generating Reactive Oxygen Species (ROS) leading to lipid peroxidation (LPO) derived aldehydes, such as 4HNE that are substrates of *ALDH-III* (Figure 7b). Although ROS trigger the formation of H_2_O_2_, *FBti0019627* does not confer resistance to oxidative stress induced by H_2_O_2_. This suggests that the effect of the insertion is mediated by *ALDH-III*. (C) Benzaldehyde and carbofuran are likely selective agents driving the differences in survival rate, and thus the increase in *FBti0019627* frequencies in natural populations of *D. melanogaster*. Each horizontal line represents one haplotype. The red box represents *FBti0019627* insertion and the blue dots represent other mutations. The pie charts show the frequency of flies with the TE (red) and without the TE (black) [16].

Mapping genotype to phenotype is currently one of the key challenges in biology [52]. Our approach to genotype-phenotype mapping in *D. melanogaster* combines a genome-wide screen for adaptive TE insertions, in which we gathered several lines of evidence suggesting their adaptive role, with hypothesis-driven mechanistic and functional analyses of the identified TEs [16], [17], [20], [22]. In this study, we further showed that this approach is able to identify true biological signals of selection and to provide a causal link between genotype and phenotype. By integrating results from gene structure and gene expression analyses, we were able to identify the molecular effect of the insertion (Figure 8A). We combined these results with the wealth of genetic and biochemical information available for Drosophila to construct mechanistic models and experimentally verify their predictions (Figure 8B). Our results provide a plausible explanation for the increase in frequency of *FBti0019627* insertion in out-of-Africa populations (Figure 8C), and adds to the limited number of examples in which a natural TE insertion has been linked to its ecologically relevant phenotypic effect [21], [22], [53].

Besides the other candidate adaptive TE insertions already identified [16], [17], the increasing availability of next generation sequencing data and of computational pipelines to estimate the frequency of TEs in populations should lead to the identification of a larger set of candidate adaptive TEs in the near future [54]–[56]. The in-depth individual analysis of these TEs is very promising, and it should help us obtain a general picture of the adaptive process.

## Materials and Methods

### Fly strains

#### Outbred populations

Four outbred populations, two homozygous for the presence and two homozygous for the absence of *FBti0019627* were created in the lab by mixing inbred lines from the Drosophila Genetic Reference Panel (DGRP; [11]. The present outbred-1 population was created by mixing RAL-40, RAL-177, RAL-405, RAL-461, and RAL-908; the absent outbred-1 was created by mixing RAL-21, RAL-75, RAL-383, RAL-441, and RAL-855. The present outbred-2 population contained flies from RAL- 776, RAL-801, RAL-802, RAL-822, and RAL-894; the absent oubred-2 population contained flies from RAL-88, RAL-195, RAL-716, RAL-820, and RAL-857. For each one of these four populations, we placed ten virgin females and ten males of each strain in a fly chamber; each outbred population was maintained by random mating during 10 (oubred-1) and 5 (outbred-2) generations before starting the experiments. The census size of each population was n≈800 per generation.

#### DGRP inbred strains

We also used four individual inbred strains from the Drosophila Genetic Reference Panel [11] to perform the phenotypic assays. Strains RAL-783 and RAL-810 are homozygous for the presence of *FBti0019627* insertion. Strains RAL-391 and RAL-857 are homozygous for the absence of *FBti0019627*.

#### Introgressed strains

We created two introgressed stocks, one homozygous for the presence of *FBti0019627* and the other homozygous for the absence. We individually crossed virgin females flies from stock RAL-783, homozygous for the presence of *FBti0019627*, with male flies from stock RAL-716, homozygous for the absence of the element. F_1_ virgin females were individually backcrossed to males form the RAL-716 parental stock. F_2_ virgin females were backcrossed to the parental males and after a few days were genotyped to check for the presence/absence of *FBti0019627*. Only the F_3_ progeny of crosses from females carrying the insertion were used to establish the next generation. Backcrosses were performed for 6 generations, and brother-sister mating was then established to obtain a strain homozygous for the presence and a strain homozygous for the absence of *FBti0019627*.

#### *CG11699* mutant flies

We used the stock number 16374 from Bloomington Drosophila Stock Center that contains an insertion of a P-element in the 5′ UTR of *CG11699* leading to a null or hypomorphic mutation in this gene [42].

### 3′ RACE experiments

Total RNA was extracted form 40 mg of embryos, 50 L3 larvae, 40 5-day-old males, 40 5-day-old females, and ovaries from 35 5-day-old females using Trizol and a PureLink RNA Mini kit (Ambion). RNA was treated on-column with DNase I (Invitrogen). Reverse transcription was carried out using 3 µg of total RNA for embryos, females, and larvae and 1.5 µg of total RNA for males and ovaries. cDNA was constructed using the SuperScript II RT First Strand Synthesis system for RT-PCR (Invitrogene). We amplified the cDNA 3′ends of *CG11699* and *Kmn1* genes using a Universal Amplification primer and two nested gene specific primers for *CG11699* (5′-AGCCGCACCGATTTCGAGAGTCT-3′ and 5′-CTGGCAGCCTGGAACGAGGAATA-3′) and *Kmn1* (5′-CATGATGGAGCTGCAGTGCAATA-3′ and 5′-CCAACGGTGACCCTAAGCTATGC-3′).

The 3′ RACE products were cloned using TOPO TA Cloning Kit for Sequencing (Invitrogene) following the manufacturer's instructions. When there were several 3′ RACE products, DNA from each individual band was extracted from the agarose gel before the cloning reaction. Several clones per 3′ RACE reaction were sequenced in both directions using M13 forward and reverse primers.

### Polyadenylation Signal (PAS) and Downstream Sequence Element (DSE) motifs search

Position-specific scoring matrices were derived from the empirical analysis of *D. melanogaster* Polyadenylation Signal (PAS) and GU-Rich Downstream Element (DSE) motifs published in [25]. The log-likelihood matrix was computed assuming all nucleotides were equiprobable. Sliding windows of 6 bp were run along the 50 bp region upstream of the cleavage site to search for the occurrence of PAS motifs. Sliding windows of 7 bp were run along 50 bp region downstream of the cleavage site to search for occurrence of DSE motifs. We expected the PAS signal to be located between the nucleotide positions −26 to −12 upstream of the cleavage site and the DSE to be located between the positions +1 and +25 downstream of the cleavage site. The highest scoring motifs located in these regions were considered as the most probable PAS and DSE motifs being used.

### Total and transcript specific qRT-PCR

Total RNA was extracted from three biological samples of 50 adult males and 50 adult females (4–6 days posteclosion) using Trizol reagent and PureLink RNA Mini kit (Ambion). RNA was then treated on-column with DNase I (Thermo) during purification, and then treated once more after purification. Reverse transcription was carried out using 500 ng and 300 ng of total RNA for females and males respectively using Anchored-oligo(dT) primer and Transcriptor First Strand cDNA Synthesis Kit (Roche). The resulting cDNA was used for qRT-PCR with SYBR green master-mix (BioRad) on an iQ5 Thermal cycler.

Total expression was measured using a pair of primers specific to a 118 bp cDNA amplicon spanning the exon2/exon3 junction of *CG11699* present in both transcripts (5′-CTGGAAGCTATCCGGAGCCAA-3′ and 5′-CGTGAGACTCTCGAAATCGGTGCG-3′). Long 3′UTR isoform expression was measured using a pair of primers specific to a 91 bp cDNA amplicon located in the 3′ most region of CG11699 3′UTR and therefore, only present in the long transcript (5′-ACCAGAACATAAAACGAAACCTTTG-3′ and 5′-TGACCGAAACAAATGAAAACCG-3′). In both cases, expression was normalized using *Act5C* as an endogenous control gene (5′-GCGCCCTTACTCTTTCACCA-3′ and 5′-ATGTCACGGACGATTTCACG-3′). We used serial dilutions of plasmid DNA to derive standard curves for each amplicon. Each curve was then used to determine the quantity of the corresponding transcript relative to the reference gene taking into account the reaction efficiency of each primer pair in order to avoid spurious results caused by differences in the efficiency of the different primer pairs. Reaction efficiencies ranged between 91,4% and 99.7% (r^2^ larger than 0.99).

### Protein extract preparation

Three replicates of 30 4-to-6 day old outbred females with and without the insertion were transferred to 1.5 ml microcentrifuge tubes under light CO_2_ anesthesia. Flies were homogenized with 1 ml of cold buffer (0.22 M sucrose, 0.12 M mannitol, 1 mM EDTA and 10 mM tricine, pH 7.2) [57] using a 2 ml glass tissue grinder on ice. The homogenate was briefly centrifuged at 4°C to pellet down whole cells and other debris. The supernatant was transferred to a clean microcentrifuge tube and centrifuged for 30 minutes at 13,000 rpm at 4°C in order to obtain a pellet enriched in membranes. The pellet was re-suspended in 1 ml of membrane-disrupting homogenization buffer containing 1% Triton X-100, incubated for 15 minutes on ice, and centrifuged again for 30 minutes at 13,000 rpm at 4°C (adapted from [58], [59]). The supernatant, containing the solubilized proteins that were bound to the membrane, was transferred to a clean microcentrifuge tube and was immediately used for protein quantification and enzymatic activity determination.

### *ALDH-III* enzymatic activity determination

*ALDH-III* (EC 1.2.1.5) oxidizes benzaldehyde to benzoic acid using NAD(P)+ as an acceptor and producing NAD(P)H in the process. We measured *ALDH-III* activity by monitoring the increased in absorbance at 340 nm produced by the formation of NAD(P)H (Figure S2A). To control for differences in overall protein abundance between samples, we quantified the total protein content for each homogenate using Quick Start Bradford Protein Assay (BioRad) following the manufacturer's instructions. Each enzymatic activity determination was performed by mixing approximately 100 µg of membrane protein extract (100–200 µl) with 1 ml of reaction buffer (50 mM sodium phosphate, 1 mM NAD+, 1 mM NADP, pH 8 and benzaldehyde at 0.01, 0.05, 0.1, 0.5 or 1 mM) in 1.5 ml disposable cuvettes. Negative controls without substrate and negative controls without NAD(P)+ were run to ensure that the formation of NAD(P)H was specific for the assay conditions we wanted to test. We measured the formation of NAD(P)H every minute at 340 nm for 15 minutes using a UV-1700 PharmaSpec spectrophotometer (Schimadzu). The slope of the linear increase in absorbance over the measurement time for each condition (R^2^>0.98) was used as initial reaction rate. Once we confirmed that *ALDH* activity showed a linear relationship with the total amount of protein used in the assay (Figure S2B), we normalized the enzymatic activity measures by the total amount of protein in each sample (activity was expressed as mOD·min^−1^·mg^−1^).

In order to estimate Vmax in our samples, we fitted the Michaelis-Menten equation to our experimental data by least-squares method using GraphPad Prism version 6.0e for Mac OS X (GraphPad Software, La Jolla California USA). We performed a *replicates test for lack of the fit* and obtained a p-value greater than 0.05, indicating that there was no evidence to reject the Michaelis-Menten model. We estimated Km and Vmax and their corresponding 95% confidence intervals and performed a statistical analysis of the comparison between the three replicates of flies with and without the insertion.

### Homology based modeling of *D. melanogaster ALDH-III* protein

The *D. melanogaster* protein sequence of *ALDH-III* isoform Q (NP_724562.2) was used to search for a structurally resolved closely related protein. The structure of human *ALDH3A1* co-crystallized with *aldi1*, a covalent inhibitor of *ALDH3A1*, was selected as a template (PDB ID: 3SZB). The alignment of these two protein sequences was built using the *pfam* hidden markov model of *ALDH* family (*Aldedh*) and the package *hmmer3/b 3.0*. The final alignment had a 51.54% identity along 424 amino acids comprising the catalytic domain, the NAD binding domain, and part of the bridging domain of *ALDH-III*.

The *ALDH-III* model was built using the automodel class in *Modeller 9.7* [60]. Energy minimization was carried out using *VMD* [61] and the extensions *Automatic PSF builder* and *NAMDgui* [62]. Default parameters were used except for the dielectric constant, which was set at 80 to simulate an implicit water environment. The stereochemical properties of the template and the model were evaluated using *PROCHECK* [63] and their pseudoenergetic profiles and z-score were calculated and compared using *PROSA-II* [64]. Superimposition of the model with human *ALDH3A1* and retrieval of the corresponding structural alignment was performed using *STAMP* [65]. For the regions were the pseudo-energy in the model was higher than in the template, the *PSI-PRED* [66] predicted secondary structure of the model was compared with the description obtained using *dssp* [67].

### Docking simulations

The docking simulations were performed for a known *ALDH-III* inhibitor, *aldi1*, and for carbofuran [68]. The protein structures and ligands were prepared for docking using the Autodock plugin for PyMol [69]. The energy-scoring grid was prepared as a 20 Å×20 Å×20 Å box centered around the catalytic cysteine of the *ALDH-III* model. The obtained ligands and receptor were used as the input for *vina* with default parameters [70]. The docking results were visualized and evaluated using PyMol. Redocking of *aldi1* with human *ALDH3A1* was used to verify that the docking parameters specified for this docking study were correct. The 10 highest scoring poses of 5 docking simulations were evaluated by comparing their localization in the catalytic pocket with the localization of *aldi1* in a structural superimposition of *ALDH-III* model and *ALDH3A1*. The criteria that were used to select the best poses were: (i) co-planarity of the aromatic rings with Tyr-114 and *aldi1*; (ii) orientation of the carbamate group towards Cys-243; and (iii) distance between the carbamate electrophilic carbon and nucleophilic thiol group of the active site cysteine.

### Phenotypic assays

#### Acute exposure to benzaldehyde

50 4-to-6 day-old females and males were placed in 50 ml fresh food vials the day before the acute exposure to benzaldehyde experiment was performed to allow flies to recover from the CO_2_. Three (first experiment) or nine (second experiment) vials of 50 individuals each per sex and per strain were exposed for five minutes to 10 µl of benzaldehyde (Sigma Aldrich) deposited on a cotton swab. Three consecutive exposures separated by 3-hour intervals were performed to each vial as reported in Arthaud et al (2011) [29]. The number of dead flies was counted 72 hours after the last exposure. As a negative control, flies were exposed to a cotton swab with 10 µl of water and the number of dead flies was also counted after 72 hours.

#### Carbofuran resistance assay

We first performed a dose-response assay using 0 µM 20 µM, 40 µM and 60 µM of carbofuran (PESTANAL, Sigma Aldrich) that was added to the food at 40°C to avoid loss of activity of the insecticide. For each carbofuran concentration, we performed 15 replicas with 10 flies per vial containing 4 ml of food each. Once we established the dose-response, we placed 20 8-to-10 day old males and females per vial, 20 replicas per sex and per strain, in food containing 60 µM of carbofuran. Flies were maintained at room temperature and dead flies were counted one to four times a day until the end of the experiment. Experiments were always done simultaneously for control flies to rule out other sources of variations in viability.

#### H_2_O_2_ resistance assay

We performed a dose-response assay using 0%, 0.25%, 0.50%, and 1% of H_2_O_2_ (35 wt. % in H_2_O, Sigma Aldrich). For each H_2_O_2_ concentration, we performed 15 replicas with 10 flies per vial containing 4 ml of food each. The experiment was then performed with a 1% final concentration of H_2_O_2_. 20 8-to-10 day old flies were placed per vial and 20 replicas per sex and per strain were performed. Flies were maintained at room temperature and dead flies were counted one to four times a day until the end of the experiment. Experiments were always done simultaneously for control flies to rule out other sources of variation in viability.

## Supporting Information

Figure S1 *FBti0019627* insertion affects *CG11699* isoform abundance. Transcript specific Real-Time PCR quantification of *CG11699* transcripts was performed using a pair of primers common to all the transcripts (total) and a pair of primers specific to the 3′ most distal region of the long 3′UTR isoform (long). Average copy number of *CG11699* relative to *Act5C* of three biological replicas with error bars representing S.E.M in flies with and without the insertion and both for males and for females are given.
(PDF)

Figure S2 (**A**) The absorbance spectrum at minute 35 shows the formation of a NAD(P)H peak at 340 nm. The size of the peak increases with increasing concentrations of substrate (0.05 mM to 5 mM benzaldehyde). (**B**) *ALDH-III* activity shows a linear relationship with the amount of total protein used in the assay.
(PDF)
