# Supplementary figures and images for: A Transposable Element Insertion Confers Xenobiotic Resistance in Drosophila
Source: PLoS Genet. 2014 Aug 14;10(8):e1004560. doi: 10.1371/journal.pgen.1004560 (PMC4133159; doi:10.1371/journal.pgen.1004560)

Figure S1

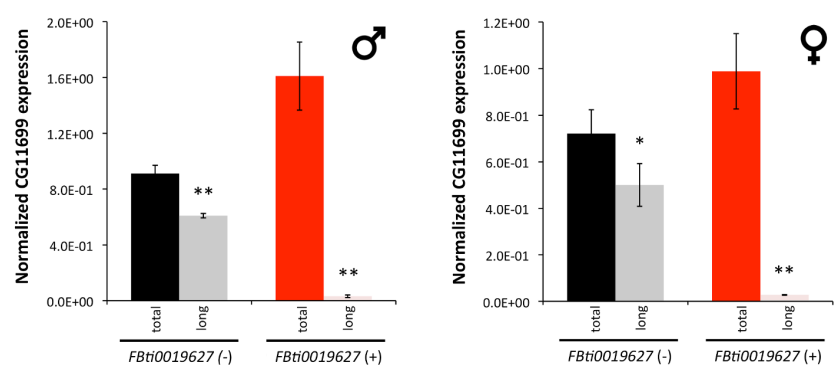

Supplement: Figure S1 — FBti0019627 insertion affects CG11699 isoform abundance. Transcript specific Real-Time PCR quantification of CG11699 transcripts was performed using a pair of primers common to all the transcripts (total) and a pair of primers specific to the 3′ most distal region of the long 3′UTR isoform (long). Average copy number of CG11699 relative to Act5C of three biological replicas with error bars representing S.E.M in flies with and without the insertion and both for males and for females are given. (PDF) [file pgen.1004560.s001.pdf]

Figure S2

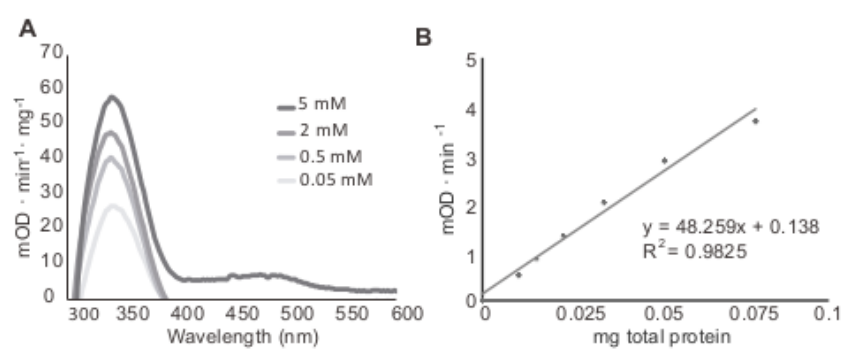

Supplement: Figure S2 — (A) The absorbance spectrum at minute 35 shows the formation of a NAD(P)H peak at 340 nm. The size of the peak increases with increasing concentrations of substrate (0.05 mM to 5 mM benzaldehyde). (B) ALDH-III activity shows a linear relationship with the amount of total protein used in the assay. (PDF) [file pgen.1004560.s002.pdf]
